# The prevalence of anemia in the patients who survived and died due to myocardial infarction (MI)

**Published:** 2015

**Authors:** F Fariba, M Moradi, MA Seifrabie

**Affiliations:** * Department of Cardiology, Hamadan University of Medical Sciences, Hamadan, Iran; ** Department of Social Medicine, Hamadan University of Medical Sciences, Hamadan, Iran

**Keywords:** myocardial infarction, ischemic heart disease, anemia

## Abstract

**Introduction & Objective:** Ischemia heart disease (IHD) is the most often met reason for losses in different communities. The most common reason of IHD is Atherosclerosis, and various etiologic factors such as high blood pressure, diabetes, smoking, and hyperlipidemia are involved in its development. Anemia is also considered another resulting reason of loss and morbidity in patients via IHD. Accordingly, the current paper aimed to determine the prevalence of anemia in patients who survived and died of myocardial infarction.

**Materials and Methods:** The present research is of a case-control type, and the subjects were the patients with myocardial infarction admitted to Heart Ward of Hamadan Ekbatan Hospital within one year (2012-2013). The patients were divided into two categories of demised and survived after myocardial infarction, and in each 160-subject group, the prevalence of anemia with hemoglobin levels was investigated. Moreover, the demographic characteristics, ejection fraction, and patients’ underlying medical conditions were also taken into account. The data obtained were analyzed by SPSS 15 software and chi-square test.

**Results:** Of the 320 patients studied, 61 patients (19.1%) had anemia and 51 (83.6%) patients who were anemic had died. 31.7% of the deceased patients after MI were weak, while 6.3% of the survived patients after MI were anemic. Also, in all age and sex groups, anemia in the former patient group was higher than in the sustained group. The predominance of anemia was clearly greater in the women than in the men (P < 0.05).

**Conclusion:** Based on the findings, there is a statistically clear variation in the prevalence of anemia in the former patient group after MI than in the group who survived after MI.

## Introduction

Ischemic heart disease (IHD) is a disease in which blood and oxygen do not reach the myocardium to the extent required by the heart, in such a way that the balance between the oxygen demand and its consumption in the myocardium fails to establish. This can result from the further demand of the heart muscle to oxygen, thereby loss of blood supply to the myocardium and also its ischemia [**1**].

One of the most frequent conditions of ischemic heart disease in the world is mortality. Given the prevalence of IHD, more attention is increasingly paid to this disease, how to treat and prevent it by doctors, and researchers. Many risk factors are discussed in the context of the creation of IHD including diabetes, smoking, high-calorie diet, high blood pressure, and urban life [**1**].

Another factor that has been taken into consideration in recent years is anemia, a lot of research being performed in understanding its impact on the prognosis of patients with MI. Recent studies have shown that patients with IHD, who are also anemic, are more likely to experience severe consequences caused by ischemic attacks. It was revealed that anemia in cases who have died because of MI is more common [**2**].

In Iran, anemia has a relatively high prevalence. Iranian women are more anemic than others, yet less attention is paid to their treatment. On the other hand, although doctors have known the signs and symptoms of anemia, they have not made any serious effort to treat and track the status of their patients’ anemia. However, doctors’ more familiarity with severe complications of anemia in heart disease such as myocardial infarction (which can be life threatening), may encourage them to pay more attention to the treatment of anemia among the patients [**3**].

In some studies, anemia significantly increased mortality up to 12% within five years, and up to 44% over the age of 15. The same survey found that the relationship between anemia and mortality from myocardial infarction is not a linear relationship [**4**].

The study of Mahmudi et al., Heart Center instructors, showed that 17.9% of the patients with IHD with low hemoglobin levels identical to those of anemia suffered from a myocardial infarction, while only 8.8% of the IHD patients with standard or high hemoglobin levels suffered from myocardial infarction [**3**].

In another study by Shu and his colleagues, anemia, and diabetes as the risk factors for death were considered within 1 month after the occurrence of myocardial infarction. In the study performed on the anemic patients with MI, it was indicated that the 30-days mortality was higher in diabetic patients (about 34%) [**5**].

In the research conducted by Al-Falluji et al. anemia also had a high prevalence among the patients who were older and the treatment of anemia with other common therapies led to the faster treatment of the heart condition in these patients. The current study emphatically suggests that treatment of anemia in these patients must be prioritized [**6**].

Concerning the likely effects of anemia on mortality caused by MI, further investigation is necessary, and in the case of its high proof, the treatment of anemia should be proposed as the first treatment of cases via IHD to be able to prevent severe complications caused by it. Given the importance of the above-mentioned information, the current research aimed to evaluate the outbreak of anemia in the patients who survived and died of myocardial infarction.

## Materials and Methods

The present research is of a case-check type, and the subjects were the patients with myocardial infarction admitted to CCU of Hamadan Ekbatan Hospital within one year (2012-2013). 320 subjects were determined based on a statistical formula and through the available sampling of the statistical community. 

After doing the investigations required, the patients were categorized into two 160-persons groups of former (case group) and survived ones (control). They were also chosen according to some factors such as ejection fraction (EF LV), underlying diseases, demographic criteria, and medical records by ethical considerations, confidentiality, and also based on the following criteria. 

The anemia measurement in the study was determined by the level of hemoglobin 12 gr/ dl in males and 11 gr/ dl in females, and the type was not specified in the survey. 

MI: myocardial necrosis following ischemia with clinical symptoms of angina that was defined in the present study with ECG changes in the form of ST segment elevation associated with increased CPU-MB greater than 24 gr/ dl in the blood.

Survival criterion: the patients with primary diagnosis of complications MI, who survived after treatment.

Death rule: the patients with a primary diagnosis of MI, who died due to some causes other than the complications of MI. The diagnosis of MI cases who died because of other complications of MI, and excluded from this study.

The data obtained were analyzed by using the questionnaire developed by SPSS 15 software and by using descriptive statistics indicators and chi-square test.

## Results

The results showed that among the 320 patients studied, 92 (28.8%) were females, and 228 (71.2%) were males. 159 (49.7%) patients survived and 161 (50.3%) died. According to the criteria used in this study, 61 patients (19.1%) were anemic, and 259 patients (80.9%) were not anemic (**Table 1**).

In the current research, the youngest victim was 35 years old, and the oldest patient was 110 years old. The average age of the cases was 65.48 within the standard deviation.

The patients were divided into five groups cases according to age. Only two people died in the group 50-35 years (n = 37). 40 patients died (36.03%) in the group 51-65 years (n = 111), and 17 of them (42.5%) were anemic. In the same age group, out of 71 surviving patients (63.97%) only 6 (8.5%) of were anemic, which indicated a lower prevalence of anemia compared with the patients who died in this age team (P = 0.001).

In the age team 80-66 years, which included 143 patients, 92 patients (64.33%) died, and 51 (35.67%) also survived. The highest proportion of dead people to sustained people observed in this group was comparable to other study groups. In this age group of surviving patients, 14 (7.7%) were anemic. In this age group, 22 patients (23.9%) of those who died were anemic, which statistically indicated a high degree of anemia in patients who died than in surviving patients in this age group (P = 0.017).

In the 81-95-years group, which included 27 patients, four patients (14.82%) survived and 23 patients (85.18%) died. None of the escaped patients had anemia in this age group, while 8 cases (34.8%) of the patients who passed away in this age group were anemic (P = 0.16). In the age group over 95 years, there were only two patients, and every two people died. Also, due to their advanced age and underlying medical condition, they were not considered reliable data, while each had 2 cases of anemia.

**Fig. 1:**
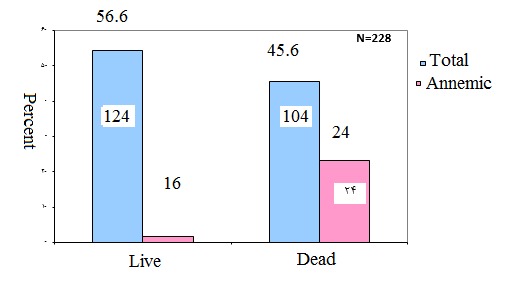
Comparison of anemia prevalence in the cases who survived and were lost because of MI in males

Of the males who survived, only two patients (1.6%) were anemic. Of the male people who died, 24 (23.1%) had anemia. In this group, anemia was statistically higher than in the surviving group (P = 0.001) (**Fig. 1**).

**Fig. 2:**
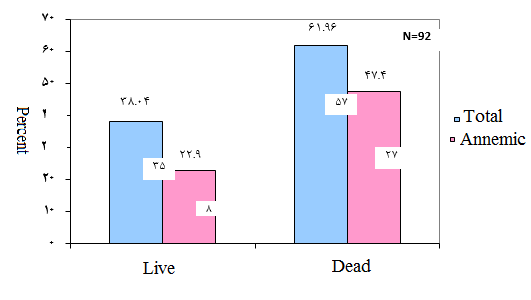
Comparison of anemia prevalence in the cases who survived and were lost because of MI in males

Among the surviving females, 8 cases (22.9%) were anemic. Also, among the female patients who died, 27 (47.4%) were anemic. In this group, anemia was mainly larger in the dead patients in comparison with the patients who survived (P = 0.019) (**Fig. 2**).

Of the patients who were surveyed, 121 patients (37.8%) had an ejection fraction lower than 50% (62.2%), and 199 (62.2%) had an ejection fraction larger than 50%. Among the patients who survived in the group with the ejection fraction more than 50%, five patients had anemia, while 20 patients among the dead patients had anemia, this being indicative of the higher proportion of anemia in the group who died than in the surviving team (P = 0.001).

In the team with an ejection fraction of less than 50%, representing the patients who had anemia, 86.1% died, and only 13.9% survived. In this group, anemia was statistically significant more frequent among the patients who died than in patients who survived in the same group (P = 0.013).

**Table 1:** Anemia prevalence in cases who survived and died due to MI

	Anemic		Non-anemic		Total	
	Number	Percent	Number	Percent	Number	Percent
Survived	10	6.3	149	93.7	159	49.7
Died	51	31.7	110	68.3	161	50.3
Total	61	19.1	259	80.9	320	100

**Table 2:** Prevalence of anemia in patients who survived and died due to MI based on higher ejection fraction and less than 50%

	Anemic		Non-anemic		Total	
	Number	Percent	Number	Percent	Number	Percent
Ejection fraction over 50%						
Survived	5	20	118	67.8	123	61.8
Died	29	80	56	32.2%	76	38.2
Total	25	12.56	174	87.44%	199	100
Ejection fraction less than 50%						
Survived	5	13.9	31	36.5	36	29.75
Died	31	86.1	54	63.5	85	70.25
Total	36	29.75	85	70.25	121	100

## Discussion 

The outcomes of this research indicated that anemia had a prevalence of about 1.19% among the cases via MI. The study conducted by Patel showed that the anemia prevalence in patients with MI was about 40.4% [**7**]. In another study conducted by Silveira, the anemia prevalence in the cases via MI was about 23%. The same survey suggested that anemia in these patients must be treated seriously [**8**]. These findings indicated that the anemia prevalence in the current research is lower than in others. The high anemia prevalence in cases via MI may have multiple causes. The patients are mostly elderly people who have underlying conditions other than heart disease. Also, anemia can also cause symptoms of ischemia in these patients.

In the current research, anemia was seen in 35 patients (38%) of the women with MI, and 26 patients (11.4%) of the men with MI, this result being reasonable taking into consideration the higher prevalence of anemia in women. Meanwhile, 47.4% of the women with MI, who were anemic, died, while 23.1% of the men with MI, who were anemic, died. It seems that anemia in women is more common, and it is more efficient in female mortality as well.

In the research performed by Madhavan et al., anemia significantly increased mortality up to 12% within five years, and 24% within a decade, and 44% within 15 years [**4**]. Although the current study did not consider the prognosis of patients during the years following MI, at first glance, a greater proportion of anemic patients with MI died.

In the research performed by Mahmoudi et al., 17.9% of the patients with IHD and anemia died, while only 8.8% of the patients with IHD with an average hemoglobin level died [**3**]. 

In the study of Zeidman et al., the mortality of the patients with MI, who were anemic was 13%, and that of non-anemic patients was 4% [**9**]. In the current study, 31.7% of the MI patients who died were anemic, while only 3.6% of the surviving patients with MI were anemic. According to the research performed in this area, anemia can significantly affect the prognosis of patients with MI. Most studies have suggested a simultaneous examination and treatment of the patients’ anemia status [**9**,**10**].

In the current research, the highest number of patients were in the age group 66-80 years, and also the maximum prevalence of anemia (29.6%) was in the age group 81 to 95 years. The highest rate of the deceased people who had anemia was seen in the age group 51-65 years. Given the prevalence of anemia in the elderly as well as the prevalence of ischemic heart diseases in the same people, knowledge and awareness of the impact of anemia on the occurrence of illness and mortality are of high importance. 

Anemia decreases oxygen-carrying capability of blood, which is the primary cause of damage to the heart muscle, and its complications include ischemia or lack of oxygen. In a study by Muzzarelli and colleagues, for a the reduction with 1 gr/ dl of the hemoglobin level, the risk of death after myocardial infarction was increased to 34%, and also the potential for other heart complications increased up to 28%, compared to the non-anemic patients with MI. In Muzzarelli’s study, same as in the current research, the type of anemia was not specified, and only the level of hemoglobin was used to determine anemia [**11**].

## Conclusion

Given the results obtained in the current study, a statistically main variation was seen in the anemia prevalence in the group who died after MI compared to the anemia prevalence in the team who survived after MI. Also, given the higher frequency of anemia in the deceased people, it is recommended that patients with IHD are checked for anemia and the anemia ones are treated to reduce the mortality and morbidity rates.

**Acknowledgments**

This paper is part of the doctoral dissertation presented at Hamadan University of Medical Sciences. The authors respectfully appreciate the devoted collaboration and efforts made by Mohammad Sa'id Besanjideh.
